# Combined radiotherapy and concurrent tumor treating fields (TTFields) for glioblastoma: Dosimetric consequences on non-coplanar IMRT as initial results from a phase I trial

**DOI:** 10.1186/s13014-020-01521-7

**Published:** 2020-04-19

**Authors:** N. Guberina, C. Pöttgen, S. Kebir, L. Lazaridis, C. Scharmberg, W. Lübcke, M. Niessen, M. Guberina, B. Scheffler, V. Jendrossek, R. Jabbarli, D. Pierscianek, U. Sure, T. Schmidt, C. Oster, P. Hau, A. L. Grosu, M. Stuschke, M. Glas, Y. Nour, L. Lüdemann

**Affiliations:** 1 Department of Radiotherapy, West German Cancer Center, University Hospital Essen, University of Duisburg-Essen, Hufelandstraße 55, 45147 Essen, Germany; 2 Division of Clinical Neurooncology, Department of Neurology and West German Cancer Center, University Hospital Essen, University of Duisburg-Essen, Hufelandstraße 55, 45147 Essen, Germany; 3 DKFZ-Division Translational Neurooncology at the West German Cancer Centre (WTZ), German Cancer Consortium (DKTK), Partner Site University Hospital Essen, University of Duisburg-Essen, Duisburg, Germany; 4 German Cancer Consortium (DKTK), Partner Site University Hospital Essen, Essen, Germany; 5 Institute of Cell Biology (Cancer Research), University Hospital Essen, Essen, Germany; 6 Department of Neurosurgery, University Hospital Essen, University of Duisburg-Essen, Essen, Germany; 7 Department of Neurology and Wilhelm Sander-NeuroOncology Unit, Regensburg University Hospital, Regensburg, Germany; 8 Department of Radiation Oncology, University Hospital Freiburg, Freiburg im Breisgau, Germany; 9 German Cancer Consortium (DKTK) Partner Site University Hospital Freiburg, Heidelberg, Germany

**Keywords:** Dosimetry, Non-coplanar IMRT, Glioblastoma, TTFields

## Abstract

**Background:**

Glioblastoma is a rapidly proliferating tumor. Patients bear an inferior prognosis with a median survival time of 14-16 months. Proliferation and repopulation are a major resistance promoting factor for conventionally fractionated radiotherapy. Tumor-Treating-Fields (TTFields) are an antimitotic modality applying low-intensity (1-3 V/cm), intermediate-frequency (100-300 kHz) alternating electric-fields. More recently interference of TTFields with DNA-damage-repair and synergistic effects with radiotherapy were reported in the preclinical setting. This study aims at examining the dosimetric consequences of TTFields applied during the course of radiochemotherapy.

**Methods:**

Cone-beam-computed-tomography (CBCT)-data from the first seven patients of the PriCoTTF-phase-I-trial were used in a predefined way for dosimetric verification and dose-accumulation of the non-coplanar-intensity-modulated-radiotherapy (IMRT)-treatment-plans as well as geometric analysis of the transducer-arrays by which TTFields are applied throughout the course of treatment. Transducer-array-position and contours were obtained from the low-dose CBCT’s routinely made for image-guidance. Material-composition of the electrodes was determined and a respective Hounsfield-unit was assigned to the electrodes. After 6D-fusion with the planning-CT, the dose-distribution was recalculated using a Boltzmann-equation-solver (Acuros XB) and a Monte-Carlo-dose-calculation-engine.

**Results:**

Overdosage in the scalp in comparison to the treatment plan without electrodes stayed below 8.5% of the prescribed dose in the first 2 mm below and also in deeper layers outside 1cm^2^ at highest dose as obtained from dose-volume-histogram comparisons. In the clinical target volume (CTV), underdosage was limited to 2.0% due to dose attenuation by the electrodes in terms of D95 and the effective-uniform-dose. Principal-component-analysis (PCA) showed that the first principal-position-component of the variation of repeated array-placement in the direction of the largest variations and the perpendicular second-component spanning a tangential plane on the skull had a standard deviation of 1.06 cm, 1.23 cm, 0.96 cm, and 1.11 cm for the frontal, occipital, left and right arrays for the first and 0.70 cm, 0.71 cm, 0.79 cm, and 0.68 cm, respectively for the second-principal-component. The variations did not differ from patient-to-patient (*p* > 0.8, Kruskal-Wallis-tests). This motion led to a diminution of the dosimetric effects of the electrodes.

**Conclusion:**

From a dosimetric point of view, dose deviations in the CTV due to transducer-arrays were not clinically significant in the first 7 patients and confirmed feasibility of combined adjuvant radiochemotherapy and concurrent TTFields.

PriCoTTF Trial: A phase I/II trial of TTFields prior and concomitant to radiotherapy in newly diagnosed glioblastoma.

DRKS-ID: DRKS00016667.

Date of Registration in DRKS: 2019/02/26.

Investigator Sponsored/Initiated Trial (IST/IIT): yes.

Ethics Approval/Approval of the Ethics Committee: Approved.

(leading) Ethics Committee Nr.: 18–8316-MF, Ethik-Kommission der Medizinischen.

Fakultät der Universität Duisburg-Essen.

EUDAMED-No. (for studies acc. to Medical Devices act): CIV-18-08-025247.

## Background

Glioblastoma (World Health Organization grade IV glioma [1]) is a rapidly proliferating tumor [2, 3]. Despite a trimodal approach, overall survival and progression free survival rates remain low [4–8]. Proliferation and repopulation are a major resistance promoting factor for conventionally fractionated radiotherapy [9]. Therefore, multimodal treatment is at present regarded as the best approach for handling this most common, aggressive brain tumor entity of adulthood.

Alongside with other established treatment options for glioblastoma (GBM) patients such as radiotherapy, surgical resection and temozolomide (TMZ) chemotherapy [10], Tumor Treating fields (TTFields) are a recently established modality reported as an effective maintenance therapy prolonging progression-free, overall and long-term survival when applied after radiochemotherapy together with TMZ in a positive phase III trial [11]. Though formally regarded as a negative trial, TTFields showed some effectiveness in recurrent glioblastomas [12]. In GBM patients TTFields therapy at 200 kHz (Optune®) (manufacturer: Novocure GmbH, Munich, Germany) is delivered by four transducer arrays that are applied to the patients scalp. Each transducer array is composed of 9 ceramic discs having very high capacity covered by hydrogel. The discs are connected by a flexible printed circuit board for better adaptation to the head curvature. Each array contains 8 temperature sensors that monitor skin temperature. Optune® was granted marketing authorization by the FDA in 2015 and is CE marked approved [13]. TTFields act antimitotic by applying low intensity (1–3 V/cm), intermediate frequency (100–300 kHz) alternating electric fields to treat solid tumors [14, 15]. More recently, interference of TTFields with DNA damage repair and synergistic effects with radiotherapy were reported in the preclinical setting [16–18]. Moreover, first experiences were reported on clinical application of TTFields in combination with radiotherapy. The phase I trial conducted by Grossman et al. in which transducer arrays were removed during application of radiotherapy, provided first indication that the combined therapy is feasible and safe [19].

Mechanisms of interaction with radiotherapy are inhibition of proliferation by a maximal absorption of induced power during the mitotic furrow [14, 20] as well as suppression of double strand break repair by thwarting homologous recombination and by down-regulation of genes within the BRCA1 pathway genes [16, 21]. In addition, TTFields delay DNA damage repair following radiation treatment of glioma cells [16]. This led to the hypothesis that the maximum interaction between TTFields and radiation may be achieved by simultaneous application [15, 21]. Until now, there exist no data from clinical trials, which examined the simultaneous application of TTFields and concurrent radiochemotherapy after surgery. Therefore, the multicenter PriCoTTF phase I trial (European database on medical devices (Eudamed) CIV 18–08-025247) has been initiated to analyze the feasibility and safety of radiochemotherapy concomitant to TTField treatment in a first step.

Here, we report on the dosimetric consequences of transducer arrays by which TTFields are applied during the course of radiotherapy in the first seven patients of that trial. Endpoints of analysis were the accumulated dose during the course of radiotherapy in the clinical target volume (CTV) and in the whole brain outside the target volume as well as in the skin, subcutaneous tissue and calvarial bone below the transducer arrays. In addition, the variation of position of the regularly replaced transducer arrays on the skin was analyzed over the radiotherapy series.

## Methods

In a prospective study design the first seven patients of the PriCoTTF phase I trial (European database on medical devices (Eudamed) CIV 18–08-025247) treated at the University Hospital Essen between 10.07.2019 and 15.12.2019 were included in the present dosimetric analysis.

### Patients

Patients with a newly diagnosed, histologically confirmed glioblastoma were eligible for study arm A with an age ≤ 70 years and a Karnofsky performance status (KPS) ≥ 60% and for study arm B at an age > 70 years and a KPS ≥ 50%. Written informed consent was obtained from all patients prior to therapy. The local Ethics Committee approved the trial.

### Trial design

This is a prospective, open-label, non-randomized, multi-center (four sites) phase I/II trial. In study arm A, a maximum of 20 patients will be enrolled. In study arm B, a maximum of 13 patients will be included.

The primary endpoint of this phase I trial is the frequency of a set of predefined TLTs (treatment limiting toxicities), i.e. predefined treatment-limiting skin toxicities or any other toxicity expected to be related to the combination of radiotherapy and TTFields, leading to compliance rate to TTFields therapy of below 50%, assessed weekly during treatment and up to 4 weeks after the end of radiotherapy. One of the secondary endpoints is the estimation of the delivered cumulative dose distribution over the treatment series for each patient from the kV-image guidance data and comparison with the planned dose distribution. Dose deviations by more than 3.5% in more than 1 cm^3^ within the PTV or by more than 5% in less than 1 cm^3^ within the PTV will be considered as relevant. The trial will be stopped, if treatment-limiting toxicities occur in three or more patients among the first seven patients in arm A and two or more patients in arm B, respectively. Otherwise, the recruitment aim is a total of 20 and 13 patients in arms A and B, respectively. Concomitant and sequential chemotherapy was administered following institutional standards and after interdisciplinary tumor conference consensus.

### TTFields-therapy concomitant to radiochemotherapy

TTFields therapy was initiated approximately one to 2 week before radiotherapy. Patients were trained on handling the device by a support specialist from the manufacturer. In addition, the treating physician advised patients to use TTFields at least 75% of the time. Positioning of the four transducer arrays consisting of nine electrodes with a diameter of 2.0 cm on an adhesive tape was planned by the manufacturer according to routine clinical care and to the localization of the tumor. The four arrays were fixed by adhesive tapes on the bald-shaved head. The arrays were changed every three to 4 days and prior to the positioning of new arrays the skin was allowed to be uncovered from the arrays for about four to six hours. Radiotherapy treatment was performed through the transducer arrays by turning off the field generator before irradiation and turning it on immediately after irradiation. At the days of array change, patients were irradiated without the arrays, which were relocated immediately after the radiotherapy. Patients were instructed to shift the arrays from their initial position about half the electrode diameter, i.e. 1 cm, every other change of the transducer arrays.

### Radiotherapy

Radiotherapy planning was based on a 1.5 mm slice thickness, contrast enhanced computed tomography (CT) scan (Siemens Healthineers, Erlangen, Germany) and fused with the postoperative MRI-scan (Eclipse version 15.5, Varian Medical Systems, Palo Alto, CA, US). The planning CT-scan was performed without transducer arrays within the field of view at the day of array change in order to avoid beam hardening artifacts. A mask system consisting of two half-shells was mounted on a base frame that can be separated by a spacer in mm-steps up to 4 mm (BrainLab, Munich, Germany) allowing the later immobilization of the patient with affixed transducer arrays. At the end of the treatment planning session, patients relocated and fixed the TTF electrode arrays with adhesive tapes following the advice of the planning radiation therapy technologists and physicians. The CT-scan was repeated after immobilizing the patient with the mask system using a spacer.

In the case of normofractionation the maximal dose at the brain stem was planned not to exceed 54 Gy, and 55 Gy at the chiasm and the optic nerves. Using hypofractionation, the maximal dose was limited to 40 Gy at the brain stem, chiasm and optic nerve. A surface dose > 70% of the prescribed dose within or up to 6 mm below the skin on a scalp was defined as high skin dose area according to the PriCoTTF phase 1 trial protocol. Patients were classified by the high skin dose area of ≤ vs. > 50 cm^2^ at low or high risk for skin toxicity. In accordance with the EORTC-ACROP guideline the clinical target volume (CTV) comprised an additional margin of 2 cm around the GTV delineated on contrast-enhanced postoperative T1-weighted MRI sequences, as well as suspicious hyperintensities on FLAIR (fluid attenuated inversion recovery) sequences with respect to anatomical borders [22]. The planning target volume (PTV) margin was defined by an additional margin of 2–5 mm. A 6D freedom couch was necessary for a precise image guidance of a PTV margin with 2 mm margin. Radiotherapy was delivered either conventionally fractionated at 2 Gy per fraction ad 60 Gy for patients in arm A or hypofractionated at 2.67 Gy per fraction ad 40.05 Gy, 5 fractions per week, for patients in arm B. Coverage of the PTV should be ≥90% and the D98 of the PTV should be > 95%. Non-coplanar IMRT (Intensity-modulated radiotherapy) or non-coplanar volumetric modulated arc therapy was delivered with 6 MeV photons at a linear accelerator (True Beam STx, Varian Medical Systems, Palo Alto, CA) in order to reduce exit dose in the contralateral brain. The linear accelerator was equipped with six degrees of freedom (6-DoF) couch. For image guidance, a low dose cone beam CT (CBCT) was acquired before each radiotherapy fraction. The CBCT imaged volume was confined to the skull above the supraorbital line in order to avoid unnecessary irradiation of the eye lens. Clinical dose planning was performed with the Acuros XB calculation algorithm version 15.11.3 available with Aria 15.5. (TPS, Varian Medical Systems, Palo Alto, CA) using non-coplanar, static field and rapid arc IMRT at the Varian TrueBeam linear accelerator system (LINAC, Varian Medical Systems, Palo Alto, CA).

### Chemotherapy

Concomitant and adjuvant chemotherapy are not investigated in this trial and will be administered according to institutional standards and interdisciplinary tumor conference.

### Conversion of TTF composition to CT numbers for dose calculation

For accurate dose calculation with TTFields, the conversion of the electron density of the TTFields relative to water to the CT-numbers in Hounsfield units (HU) had to be established. This was done by two methods: first, by HU estimation from the material composition of the TTFields and second, by measuring the transmission factors behind the TTFields in a plastic water phantom (CIRS, Norfolk, VA, USA) with a Markus chamber (PTW-Freiburg, Freiburg, Germany). Material composition of the TTFields was determined as pertinex, ceramic, and gel. The atomic composition of the material fractions was obtained and the relative volumes and weights were measured resulting in a weighted average HU of 3817. Secondly, the depth dose curve and the measured doses in the water phantom at depth of 5 to 15 cm behind the TTFields were compared with that calculated behind a contoured TTField structure using density overrides of the TTField contours by the Acuros XB algorithm version 15.11.3 available with Aria 15.5. (TPS, Varian Medical Systems, Palo Alto, CA). The Acuros XB algorithm offers the opportunity to assign a HU from 3832 to 7484 as titanium alloys to structures. The leading HU value fitting best to the measured dose distribution in water behind the TTFields was 3832 HU, associated with a mass density of 3.56 g/cm^3^. The latter HU value was used for dose accumulation of the clinical treatment plans with electrodes positioned according to the cone-beam CT’s.

### Clinical dose calculation and accumulation

The pre-fraction cone-beam CT (CBCT) data (TrueBeam, Varian Medical Systems, Palo Alto, CA) of the 7 patients in the PriCoTTF trial were used for dosimetric verification and dose accumulation of the non-coplanar intensity modulated radiotherapy (IMRT) treatment plans. Furthermore, it served for geometric analysis of the transducer arrays throughout the course of treatment. The position of the frontal, occipital, right and left-sided array was obtained by assessing the x-, y- and z-vectors from the low-dose CBCT-scans. The x-, y- and z-vectors were determined at the center of one of the nine TTFields of each transducer array throughout all fractions. With an approximate volume of 36 cm^3^ cleared from beam hardening artifacts, transducer array structures were contoured for each fraction with the “Image Thresholding” tool (TPS Eclipse, Varian Medical Systems, Palo Alto, CA, US). Transducer contours were copied back to the planning CT using the 6 degree of freedom on-line match between the respective cone beam CT and the Planning CT. Consecutive two mm thick surface contours were delineated in the planning CT till a depth of 10 mm below the surface using the body contour and excluding all TTF structures from each fraction as the surface. For each fraction with TTFields the actual transducer arrays were integrated into the body contour and overwritten with a density characterized by 3832 HU. The individual fraction doses were calculated and accumulated over all fractions using a dose grid of 1.5 mm. Dose accumulation with TTFields over the whole treatment series was performed by adding the doses calculated in the planning CT with TTFields of the respective fraction over all fractions voxel-wise over the body volume. In addition, a difference plot was calculated for the accumulated doses with and without TTFields. Hot spots in different organs and cold spots in the CTV were examined. Dose distributions were calculated with both, a Boltzmann equation solver (Acuros XB, Eclipse version 15.5., Varian Medical Systems, Palo Alto, CA) and a Monte Carlo dose calculation engine (Prosoma, version 4.2.). The Monte Carlo dose calculation engine implemented in Prosoma version 4.2. is based on the VMC++ and XVMC- code. It relies on a virtual source model (VSM) of the linear accelerator head. The VSM applies a primary and a secondary photon source as well as an electron contamination source [23], derived from a full Monte Carlo Simulation of the accelerator head with the BEAMnrc MC system [24]. The cut-off electron energy in the Acuros XB algorithm is 200 keV compared to 240 keV in the Monte Carlo algorithm. Altogether *n* = 10^9^ primary histories were calculated per calculated dose distribution with Monte Carlo algorithm with a photon cut-off of 60 keV.

### Radiobiological models

The equivalent uniform dose (EUD) for the CTV was obtained according to the clonogen survival model [25]. The fraction of clonogenic tumor cells surviving at 2 Gy (SF2) was assumed to be 0.5445 [26]. This led to a tumor control probability of 30% for a tumor with 10^8^ clonogens at a total dose of 60 Gy with 2 Gy/ fraction. The estimate of the number of clonogenic tumor cells was obtained from Suit 1992 [27]. In addition, the fractionation sensitivity was characterized by an alpha/beta value of 10 Gy [26]. The validity of the linear quadratic cell survival model was assumed and the respective surviving fraction of 0.4244 at 2.67 Gy was obtained. As another parameter of the effectiveness of the delivered radiation dose, the D95 (minimum dose within the 5% of voxels with the highest dose in the CTV) was evaluated. As a parameter for toxicity in the brain outside the planning target volume, the minimum dose in the 2% voxels at highest accumulated dose in this structure was analyzed. As skin tolerance increases with decreasing exposed area and is above 60 Gy with conventional fractionation for areas below 30 cm^2^, we quantified skin exposure as maximum doses outside areas of 1 cm^2^ or 25 cm^2^ at highest doses [28].

### Statistical analysis

Statistical analyses were performed in SAS (version 14.1, SAS Institute, Cary, NC, US). All statistical tests and procedures used in this study are specified along with the results. The procedure PRINCOMP was used for principal component analysis. The procedure NPAR1WAY was performed for computation of the empirical distribution functions (SDF). The Kruskal-Wallis tests and Wilcoxon scores were applied for testing statistically significant differences. The procedure UNIVARIATE was used for calculation of means as well as standard deviations. Kolmogorov-Smirnov test was applied for testing normality. The given *p*-values were 2-sided, the level of significance was set at < 0.05.

## Results

### Clinical dose calculation and accumulation

**Fig. 1:**
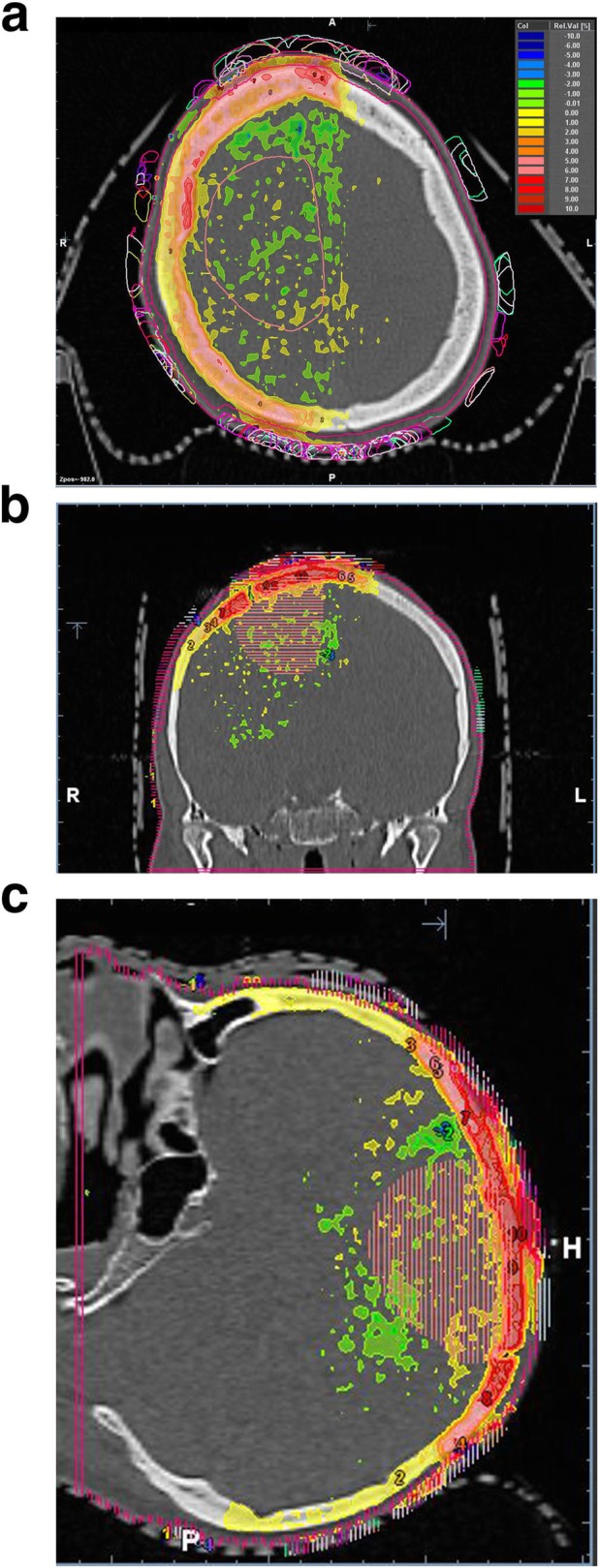
(**a-c**) Highlighting dose difference plots of accumulated dose distributions with and without electrodes (**a-c**): Delineated clinical target volume (right frontoparietal), 2mm shell contour and TTF array structures over the whole series (**a**) right: axial computed tomography; (**b**) middle: aligned, coronar reconstruction; (**c**) left: sagittal reconstruction. Dose differences are expressed as percentages of the prescribed dose

All seven patients finished concomitant treatment without interruptions or major protocol deviations. Characteristics of the prescribed treatments and details of the applied radiotherapy techniques are given in Table 1. Tables 2 and 3 summarize the dosimetric results from dose accumulation over the treatment series as captured by the cone beam CTs with TTFields and overwriting density of the TTFields with 3832 HU calculated using Acuros XB or Monte Carlo algorithm. As a measure of effectiveness, the D95 for the CTV did not decrease by more than 2% in comparison to plans without TTFields and was typically below 1%. The same applied for the equivalent uniform dose (EUD). In general, there was a very good agreement of the results by the Acuros XB and Monte Carlo algorithm. The same is valid for organs at risk as brain outside PTV (Brain – PTV) except for the patient in the fourth row of Tables 2 and 3. In this case a dose difference of 4% with and without electrodes was observed with the Acuros XB, but only of 1.2% with the Monte Carlo algorithm. Both algorithms revealed a reduced dose build-up in the first five 2 mm layers (0–10 mm) below the surface of the scalp by the electrodes. The maximum doses outside the “hottest” 1 cm^2^ at highest dose differed by less than 8.5% of the prescribed dose according to the Acuros XB and the Monte Carlo algorithm. While Tables 2 and 3 compare the dose volume histograms for the indicated structures with and without electrodes and thereby lose spatial correlation of the voxels with over- and underdosage, Table 4 shows the statistics of the observed voxel-wise dose differences in the first three, superficial 2 mm layers in the scalp for the dose difference distributions with and without TTFields. In the first 2 mm layer voxel-wise dose differences of up to 29% (range 24–29%) could be seen outside 1 cm^2^ with the largest dose differences in the build-up region determined by the Monte Carlo algorithm. However, absolute doses normalized to the prescription dose stayed below 100% outside the “hottest” 1 cm^2^. In addition, the clinical employed array-renewing schedule leading to irradiations without TTFields every third day adds to a further diminution of these over-dosages by one third. Figure 1 highlights dose difference plots of accumulated dose distributions with and without electrodes.

**Table 1:** Characteristics of the 7 glioblastoma patients: Tumor localization in the patient; study arm A or B; treatment technique; skin high dose area (surface dose > 70% of the prescribed dose within or up to 6 mm below the skin on a scalp area are of > 50 cm^2^); delivered dose; number of fractions with set up cone beam CT; temozolomide

Tumor localization in the patient identified by the respective row of table	Study arm A or B	Treatment technique	Skin high dose area	Delivered dose	Number of fractions with set up cone beam CT	Temozolomide
Left parietal	A	Non-coplanar IMRT	2.4 cm^3^/ 12 cm^2^	30 × 2 Gy	28	75 mg/m^2^
Right frontotemporal	A	Non-coplanar IMRT	4.4 cm^3^/ 22 cm^2^	30 × 2 Gy	28	75 mg/m^2^
Right parietal	B	Non-coplanar IMRT	18 cm^3^/ 90 cm^2^	15 × 2.67 Gy	5	75 mg/m^2^
Left frontal	A	Non-coplanar arcs	12 cm^3^/ 60 cm^2^	30 × 2 Gy	23	75 mg/m^2^
Right frontal	B	Non-coplanar arcs	0.4 cm^3^/ 2 cm^2^	15 × 2.67 Gy	15	75 mg/m^2^
Right parietal	A	Non-coplanar IMRT	37,1cm^3^/ 185.5 cm^2^	30 × 2 Gy	30	75 mg/m^2^
Right frontal	A	Non-coplanar IMRT	7.1cm^3^/ 35.5 cm^2^	30 × 2 Gy	30	75 mg/m^2^

**Table 2:** Acuros XB dose calculation: accumulated dose-volume characteristics for the CTV and organs at risk. Each row indicates data from a separate patient

CTV D95 without / with electrodes	CTV EUD without / with electrodes	Accumulated min surface dose to the hottest 1 cm^**2**^ in shells up to 2, 4, 6, 8, 10 mm without electrodes	Accumulated min surface dose to the hottest 1 cm^**2**^ in shells up to 2, 4, 6, 8, 10 mm with electrodes	D2 Brain - PTV without / with electrodes
100.9 / 99.7	102.4 / 101.3	69.5 / 80.0 / 88.7 / 94.5 / 102.5	77.8 / 82.0 / 87.7 / 96.3 / 100.5	83.1 / 82.5
97.9 / 99.8	100.8 / 99.9	87.5 / 99.4 /100.9 /100.5 /103.4	92.3 / 100.5 /100.5 /101.3 /103.9	88.0 / 87.0
94.4 / 93.7	97.2 / 96.6	92.1 / 97.6 / 99.0 / 98.8 / 101.0	99.9 / 99.6 / 98.9 / 98.1 / 99.3	93.4 / 92.7
96.8 / 96.3	101.0 / 100.4	97.5 /105.4 /102.5 /102.8 /106.8	101.9 /108.6 /102.9/ 102.3/ 105.6	93.8 / 97.8
98.2 / 97.5	100.1 / 99.4	82.2 / 100.5/ 102.7/ 103.6/ 104.8	88.0 / 100.2/ 101.7/ 102.6/ 103.7	74.9 / 74.9
98.4 / 97.8	101.0 / 100.3	93.8 / 103.8/ 104.6/ 105.0/ 106.3	102.3/ 104.0/ 104.5/ 104.9 /106.0	98.7 / 98.0
96.6 / 96.4	99.6 / 99.5	83.8 / 97.3 / 98.6 / 99.7 / 99.9	90.3 / 100.3/ 99.5 / 99.7 / 99.5	86.2 / 86.0

Note: doses are given as relative doses normalized to the prescribed dose; five adjacent scalp + calvaria layers were defined as tissue slices of 2 mm thickness below the surface of the scalp in a depth of 0–2 mm, 2–4 mm, 4–6 mm, 6–8 mm and 8–10 mm; D95: minimum dose in 95% most exposed voxel of the structure; D2: minimum dose in the 2% most exposed voxels of the structure

**Table 3:** Monte Carlo dose calculation: accumulated dose-volume characteristics for the CTV and organs at risk. Each row indicates data from a separate patient

CTV D95 without / with electrodes	CTV EUD without / with electrodes	Accumulated min surface dose to the hottest 1 cm2 in shells up to 2, 4, 6, 8, 10 mm without electrodes	Accumulated min surface dose to the hottest 1 cm2 in shells up to 2, 4, 6, 8, 10 mm with electrodes	D2 Brain - PTV without / with electrodes
101.6 / 100.6	103.7 / 102.4	67.8 / 80.3 / 89.1 / 96.6 / 102.8	75.3 / 86.6 / 86.3 / 86.3 / 104.4	84.1 / 83.1
98.9 / 97.9	102.1 / 101.1	87.5 / 101.7 / 106.9 / 107.5 / 107.0	93.4 / 102.4 / 107.1 /107.0 /105.9	89.3 / 88.2
95.0 / 93.7	98.1 / 97.5	93.5 / 97.4 / 99.6 / 98.9 / 100.5	98.1 / 102.0 / 106.2 / 106.8 / 105.8	94.8 / 93.8
96.3 / 95.8	101.0 / 100.3	93.5 / 103.6/ 101.5 / 101.5 / 105.4	99.0 / 110.0 / 101.5 / 109.5 / 109.9	93.0 / 92.3
97.8 / 97.5	100.6 / 100.0	80.3 / 101.3 / 107.6 / 109.2 / 109.1	87.0 / 101.5 / 107.2 / 108.0 / 107.4	74.9 / 74.5
99.4 / 98.9	102.4 / 101.5	96.6 / 102.9 / 104.3 / 104.9 / 106.3	100.3 / 106.8 / 110.0 / 110.3 / 110.8	99.4 / 98.8
100.0 / 99.2	101.8 / 100.8	82.7 / 97.7 / 98.7 / 99.2 / 99.8	89.2 / 105.6 / 109.9 / 108.3 / 107.8	87.3 /86.4

Note: doses are given as relative doses normalized to the prescribed dose; four adjacent scalp+calvaria shells were defined as tissue slices of 2 mm thickness below the surface of the scalp in a depth of 0–2 mm, 2–4 mm, 4–6 mm, 6–8 mm, and 8-10 mm

**Table 4:** Monte Carlo calculated dose build up in the scalp: dose differences from the dose differences distribution with – without TTF electrodes in layers of 2 mm thickness. Note. Dose differences are given as percentage relative doses normalized to the prescription dose

Scalp shells from 0 to 2 mm below surface		Scalp shells from 2 to 4 mm below surface		Scalp shells from 4 to 6 mm below surface	
Min dose difference in the 1 cm^**2**^ with the largest dose differences [%]	Min dose difference in the 25 cm^**2**^ with the largest dose differences [%]	Min dose difference in the 1 cm^**2**^ with the largest dose differences [%]	Min dose difference in the 25 cm^**2**^ with the largest dose differences [%]	Min dose difference in the 1 cm^**2**^ with the largest dose differences [%]	Min dose difference in the 25 cm^**2**^ with the largest dose differences [%]
24.4	12.4	11.8	6.6	13.8	7.8
29.0	14.1	13.4	6.1	8.0	3.3
26.7	11.9	17.5	7.2	17.0	9.6
27.0	11.9	13.5	6.8	16.2	9.0
26.3	9.0	10.7	3.0	5.3	1.0
27.9	15.6	13.4	9.1	14.2	10.7
24.6	7.1	13.1	5.0	13.7	8.7

**Fig. 2:**
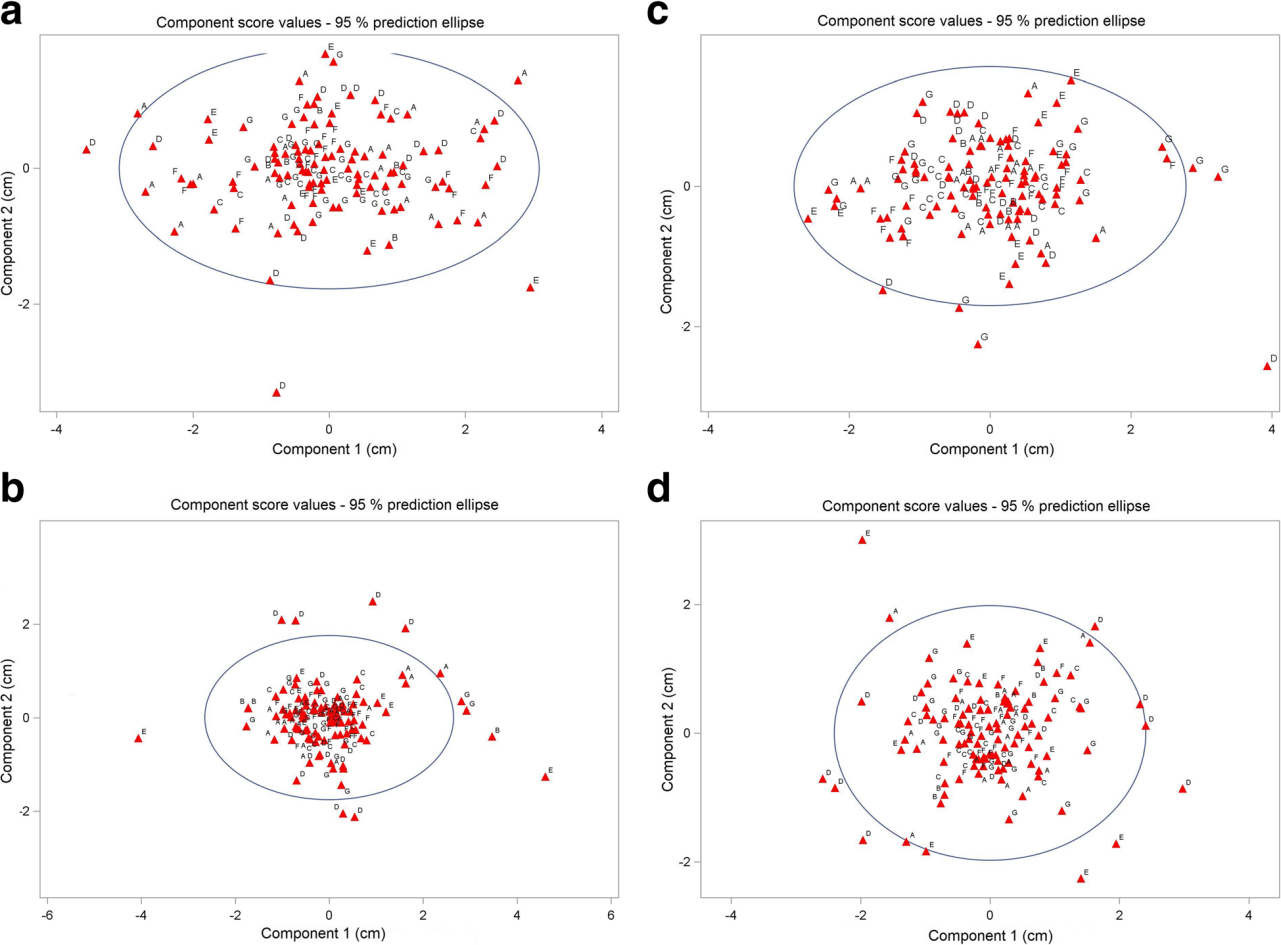
(**a-d**) Scatter plots of the first and second principal component length of the respective transducer array position deviation from overall mean position at each fraction for patients a – g (in cm): Data were obtained from pre-fraction cone beam CT’s. Data are given together with the 95% prediction ellipse for a new observation. (**a**) occipital array; (**b**) frontal array; (**c**) right side array; (**d**) left side array

Principal component analysis (PCA) showed that the first principal position component of the variation of repeated array placement in the direction of the largest variations and the perpendicular second component spanning a tangential plane on the skull had a standard deviation of 1.06 cm, 1.23 cm, 0.96 cm, and 1.11 cm for the frontal, occipital, left and right arrays for the first and 0.70 cm, 0.71 cm, 0.79 cm, and 0.68 cm, respectively for the second principal component (Table 5). Principal component score plots of the length of the first versus second principal component over all fractions and patients is given in Fig. 2 a-d for the occipital, frontal, right and left side arrays along with the 95% prediction ellipses. The normality of the distribution of the principal components was analyzed by a Kolmogorov-Smirnov test for all arrays. Some deviations were detected (Table 6). The lengths of the first principal vectors did not differ from patient to patient for all arrays (*p* > 0.8, Kruskal-Wallis tests).

**Table 5:** Principal component analysis of the placement variations of the transducer arrays: Placement variations along the principal components. Kolmogorov-Smirnov test: Test for comparison of the placement deviations from the overall mean per patient with a normal distribution

Array	First principal component		Second principal component		Third principal component	
	Standard deviation [cm]	*p*-value Kolmogorov-Smirnov test	Standard deviation [cm]	*p*-value Kolmogorov-Smirnov test	Standard deviation [cm]	*p*-value Kolmogorov-Smirnov test
frontal	1.06	< 0.01	0.70	< 0.01	0.31	< 0.01
occipital	1.23	0.01	0.71	< 0.01	0.25	< 0.01
left	0.96	> 0.15	0.79	> 0.15	0.28	< 0.01
right	1.11	> 0.15	0.68	0.02	0.25	> 0.15

**Table 6:** Principal component analysis of the placement variations of the transducer arrays: coordinates of the principal components. X, y, and z are the coordinates of a central electrode of the respective array in the CT-coordinate system relative to the electrode mean position over all fractions. Unit of length is cm

Array	First principal component [cm]/ Proportion of the variance explained [%]	Second principal component [cm]/ Proportion of the variance explained [%]	Third principal component [cm]/ Proportion of the variance explained
frontal	p1 = −0.077^.^x + 0.919^.^y + 0.386^.^z 65.7%	p2 = 0.993^.^x + 0.038^.^y + 0.110^.^z 28.8%	p3 = − 0.087^.^x - 0.392^.^y + 0.916^.^z 5.5%
occipital	p1 = − 0.058^.^x - 0.459^.^y + 0.887^.^z 72.8%	p2 = 0.997^.^x - 0.063^.^y - 0.033^.^z 24.1%	p3 = 0.041^.^x + 0.886^.^y + 0.461^.^z 3.1%
left	p1 = 0.231^.^x + 0.891^.^y - 0.390^.^z 56.8%	p2 = − 0.305^.^x + 0.447^.^y + 0.841^.^z 38.4%	p3 = 0.924^.^x - 0.076^.^y + 0.375^.^z 4.8%
right	p1 = 0.017^.^x + 0.993^.^y - 0.119^.^z 70.0%	p2 = 0.345^.^x + 0.1096^.^y + 0.933^.^z 26.3%	p3 = 0.939^.^x - 0.057^.^y - 0.340^.^z 3.7%

## Discussion

The present study was designed to investigate, in a predefined way, the dosimetric consequences of TTFields applied during the course of radiochemotherapy. The general aim was to show that radiochemotherapy can be delivered through transducer arrays, both in a practical way and with similar dose distribution as without TTFields. This is of particular importance in view of previous studies that showed that TTFields do not only induce antiproliferative and cytotoxic effects on dividing cells, but also may lead to an enhanced susceptibility and sensitivity to ionizing radiation [21]. Therefore, for the first time, our study shows from a dosimetric point of view that the translation of a combined radiochemo-TTF-based therapy into the clinical setting is feasible.

Several novelties are included in this study. First, we estimated the dosimetric effects of transducer arrays on the delivered dose distribution in patients treated with concurrent TTFields and radiation therapy. Second, we used Monto Carlo calculation for estimation of the clinically delivered dose distribution of simultaneous radiotherapy and TTFields in glioblastoma patients. In our study all treatments were performed following the specifications made by the vendor, henceforth all dose analyses were based upon these terms [13].

Previous film dosimetry measurements showed that transducer arrays may increase the dose build-up to 82, 88 and 98% of the maximal dose build-up per beam at a depth of 0.4 mm and incident beam angles on the surface of 90°, 45° and 10° [29]. In the present study, the maximal voxel-wise dose difference with and without onlying transducer arrays ranged from 24 to 29% of the prescribed dose observed in the superficial 2 mm of the scalp outside the 1 cm^2^ with the largest dose differences. The attenuation of a 6 MeV beam is about 3–4% at an incidence angle of 90° to the skin. Back scatter experiments positioning the transducer arrays at the beam exit side of the body showed an enhancement of 23% of the depth dose at the beam exit side of the body. Li et al. 2018 and Straube et al. 2018 performed treatment planning and TTF dose measurement studies in an Anderson Rando phantom [30, 31]. Either the Acuros XB v11 or the AAA13 algorithm, both implemented in the Eclipse planning system (Varian Medical Systems, Palo Alto, CA, US), were used by Li et al. 2018 and Bender et al. for dose calculation [29, 30]. Li et al. overwrote the density of the electrodes contoured on the CT of the Anderson RANDO phantom with the highest density allowed by the Acuros XB v11 algorithm, with the density of aluminium, that is lower than that of the TTFields [30]. Straube et al. used the Hounsfield units from a keV-CT and MeV-CT to estimate the electron densities of the transducer arrays [31]. Straube’s group noticed that the Hounsfield units from the keV-CT were at and above the upper limit of measurable values [31]. The MeV-CT’s tended to an underestimation of the HU by the electrodes. Li et al. found from the dose calculations that the percentage of the PTV covered by the prescribed dose, decreases by an average of 0.7% in the 10 scenarios analyzed in the phantom, but could detect dose increases in the scalp only being 2 Gy for the D1cc at maximum as a consequence of the electrodes [30]. The D1cc is the minimum dose in the most exposed 1 cm^3^, than without the electrodes from the overall dose statistic within the respective structure. In the present study, we found higher dose increases of up to 5.1 Gy for a conventional fractionated schedule up to 60 Gy in the scalp from 0 to 2 mm below the surface using the maximum dose outside the most exposed 1 cm^2^ from the dose volume histograms, corresponding to a D0.2cc at a shell thickness of 2 mm, i.e. in smaller hot spots. Straube et al. 2018 found a decrease of 1.1% in the D98 of the PTV in the analyzed scenarios similar to the results for the CTV in the present study [31]. The previous phantom studies implied that wearing transducer arrays during radiotherapy should not lead to a clinically significant underdosage of the target volume due to the attenuation of the treatment beams [30; 31]. However, increased skin doses were noticed. Skin reaction of grade III-IV is the primary endpoint of the PriCoTTF phase I trial. As a primary prophylaxis of severe skin reaction, patients were instructed to remove and replace the transducer arrays at positions differing at maximum one diameter of the electrodes around their original position.

Principal component vector analysis in our study demonstrated that the standard deviation of the position of the center electrode of the anterior and both lateral arrays is 1 cm in the direction with the largest variation, and 1.4 cm of the occipital arrays. The vector analysis showed no significant variations from patient to patient. This variation and the fact that at about each third radiation fraction, the transducer arrays were removed during irradiation, led to a marked decrease in the accumulated skin dose and the dose in the subcutaneous tissue below the electrodes.

Furthermore, we compared the clinically used Acuros XB algorithm and the Prosoma Monte Carlo algorithm used for dose verification in our department. Others have found slight differences between Monte Carlo calculations and the Acuros XB algorithm in the near the interface of water and high Z-material. Reis et al. 2019 found a good agreement in the depth dose curves in heterogeneous water phantoms with layers of bones with a density of 1.8 Gy/cm^3^ [32]. Alhakeem et al. analyzed the performance of the Acuros XB 11.0.31 algorithm in a water phantom containing a steel rood with a density of 7.8 g/cm^3^ in comparison to a Monte Carlo simulation and dosimetric measurements [33]. At the distal steel to water interface, the Acuros XB 11.0.31 algorithm underestimated the dose up to 2.8% [33]. Ojala et al. 2014 compared the agreement of Acuros XB and Monte Carlo dose calculation algorithm point dose measurements in the water phantom [34]. At the distal Ti6A14V alloy hip implant of a density of 4.42 g/cm^3^ to water interface, the Acuros XB algorithm underestimated the dose in water near the alloy surface in the shadow of the implant in comparison to the measured and Monte Carlo-calculated doses by up to 5.5% [34]. Onizuka et al. compared clinical dose distributions for head and neck patients calculated with Monte Carlo and Acuros XB v11 algorithms and found that the Acuros XB overestimated the dose in the CTV by 3–5% in comparison to the Monte Carlo simulation technique [35]. Smaller differences between Monte Carlo simulation and the Acuros XB dose calculation algorithm were observed in the superficial dose build-up region. Similar dose differences were observed downstream of the high density structures and in dose build-up regions reported by previous authors [34].

Our results confirm that the dose distribution within the CTV is not clinically significantly compromised by the transducer arrays using multifield, non-coplanar IMRT. Contrary to previous in vivo studies that examined the sequential application of radiotherapy and TTFields-treatment, our results are the first that showed that radiotherapy with concurrent TTFields-treatment offers a practical treatment option. This is of particular importance considering the fact that several studies reported synergistic effects of concurrent TTF-array treatment and radiotherapy [16, 21]. We therefore will translate these important results to an already recruiting phase I-trial. If this trial confirms overall safe of the approach and gives first signals for an increased efficacy of radiochemo-TTField-therapy, we will explore the approach further in a larger randomized trial that investigates early integration of TTFields concomitant to radiochemotherapy to the conventional sequence of radiochemotherapy followed by chemotherapy and TTField treatment.

## Conclusions

We conclude that dose deviations in the clinical target volume resulting from transducer arrays by which TTFields are applied during conformal radiotherapy treatment are with D95 and EUD differences below 2% and therefore not clinically relevant. The reduced dose build-up in the skin resulted in a dose increase of below 8.5% outside the “hottest” 1 cm^2^ at highest dose and is rated as clinically acceptable.

## Data Availability

Not applicable.

## Abbreviations

ACROP: Advisory Committee on Radiation Oncology Practice
CBCT: Cone-beam computed tomography
CE: Conformité Européenne
CT: Computed tomography
CTV: Clinical target volume
DNA: Deoxyribonucleic acid
EORTC: European Organisation for Research and Treatment of Cancer
EUD: Equivalent uniform dose
Eudamed: European database on medical devices
FDA: U.S. Food and Drug Administration
FLAIR: Fluid attenuated inversion recovery
GBM: Glioblastoma
GTV: Gross tumor volume
HU: Hounsfield unit
IMRT: Intensity modulated radiotherapy
KPS: Karnofsky performance status
PCA: Principal component analysis
PTV: Planning target volume
SF2: Fraction of clonogenic tumor cells surviving at 2 Gy
TMZ: Temozolomide
TLT: Treatment limiting toxicity
TPS: Treatment planning systems
TTFields: Tumor Treating Fields
6-DoF: Six degrees of freedom
